# On Supplementing “Foot in the Door” Incentives for eHealth Program Engagement

**DOI:** 10.2196/jmir.3701

**Published:** 2014-07-25

**Authors:** Marc Steven Mitchell, Guy E Faulkner

**Affiliations:** ^1^Faculty of Kinesiology and Physical EducationGraduate Department of Exercise SciencesUniversity of TorontoToronto, ONCanada

**Keywords:** cardiovascular risk, prevention, rewards

## Abstract

Financial health incentives, such as paying people to lose weight, are being widely implemented by Western nations and large corporations. A growing number of studies have tested the impact of incentives on health behaviors, though few have evaluated the approach on a population-scale. In this issue of the Journal of Medical Internet Research, Liu et al add to the evidence-base by examining whether a single incentive can motivate enrollment and engagement in a preventive eHealth program in a sample of 142,726 Canadian adults. While the incentives increased enrollment significantly (by a factor of about 28), a very high level of program attrition was noted (90%). The “foot in the door” incentive technique employed was insufficient; enrollees received incentives for signing-up for, but not for engaging with, the eHealth program. To supplement this technique and drive sustained behavior change, several theoretically- and empirically-based strategies are proposed. Specifically, incentives indexed to behavioral achievements over time are highlighted as one approach to boost engagement in this population in the future.

Attrition (non-use or dropout) is one of the hallmarks of eHealth interventions [1] and a particular problem for online lifestyle interventions [2], but also in the offline setting. Financial health incentives, such as paying people to attend nutrition classes, get vaccinated, or lose weight, are being widely implemented by Western nations and large corporations [3-5]. A growing number of studies have tested the impact of incentives on “single-shot” (eg, clinic visits) [6] and “lifestyle” (eg, exercise) [7]  health behaviors with promising results, though few have evaluated the approach on a population-scale. Notably, Stock et al conducted a unique evaluation of a large-scale incentive program in Germany (N=70,429) and found that modest incentives motivated participation in disease prevention programs, saving the German Statutory Health Insurance about **€** 100 per person per year in direct health costs [8]. In this issue of the *Journal of Medical Internet Research*, Liu et al add to the evidence-base by examining whether a single incentive exposure (20 loyalty points worth about Can $2) can motivate enrollment *and continued participation* in a preventive eHealth program in a sample of 142,726 Canadian adults [9].

Not surprisingly, and consistent with the “single-shot” incentives literature, the authors found that individuals offered the incentive were 28 times more likely to enroll in the eHealth program. The question is, was this impressive boost in enrollment meaningful considering the significant attrition (about 90%) noted by the authors? The data would suggest that this was the case. Although program attrition was substantial, the fact remains that about 5000 adults who might not have otherwise enrolled in the eHealth program (without the contingent incentive) continued onto the next step of the program (ie, assessing their readiness to change), and about 1000 of those individuals remained engaged 6 weeks later (ie, with a second round of assessing readiness to change).  It is not clear whether this level of engagement (assessing readiness to change on two occasions and, presumably, reading tailored-education materials) was sufficient to stimulate “lifestyle” health behaviors to the extent required to produce clinically or health-economically relevant changes. This is a topic for future study (linking participation to administrative databases, for example). Regardless, the incentive approach employed here holds great promise for enhancing the delivery (reach) of Web-based prevention programs. In the midst of this potential, however, there is room to improve upon the design, and maximize the impact, of the incentive offering. 

First, the “foot in the door” design technique employed was insufficient to drive eHealth program engagement in this population. While getting people to agree to do something small (put their “foot in the door”; ie, enroll in the eHealth program), can sometimes make them more likely to do something big (ongoing eHealth program use), according to behavioral economics [10], this is not what transpired here. Rather, it appears that “rewards simply motivated people to get rewards”, an often-cited risk of incentives for health [11]. Once incentives are removed, it is said that people tend to revert to baseline behaviors or worse, if autonomous motivation is undermined by extrinsic rewards. New evidence suggests, however, that well-designed incentives can actually promote quality behavior change (ie, by protecting/building autonomous motivation), and increase the potential for sustained effects [12].

The authors recommend supplemental strategies to maintain engagement that stem from previous work in the area of incentives for exercise [7]. From the list of design suggestions mentioned, the most critical suggestion likely has to do with indexing incentives to behavioral occurrences over time. “Lifestyle” health behaviors (like exercise or eHealth program use) can be hard to adopt, in part, because the costs (eg, time) of engaging in these behaviors are usually borne in the present (carrying disproportionate weight in decision making, a phenomenon referred to as the “present bias”) [10], and the benefits (eg, health) are often delayed and thus discounted. As a result, people tend to act in favor of their immediate self-interest, at the expense of their long-term wellbeing. Offering incentives for *regular* (perhaps daily or weekly) eHealth program use (eg, completing stage of change assessments, or diet/exercise diaries) may increase the immediately rewarding aspects of participation and in turn people’s propensity to log into and use the eHealth tool.

Regular incentive offerings need not be prohibitively costly either. In fact, with a stronger application of behavioral economic principles [13], and considering the full range of incentive design options [14], it may be possible to produce greater effects with the same investment ($77,000, or about $2 per enrollee). For example, it is worth exploring whether the proportion of Canadians engaged at 6 weeks would increase if 10 Air Miles (vs 20 Air Miles) were offered for enrollment, with the remaining Air Miles (ie, 10) offered for behavioral achievements over subsequent weeks (eg, 2 Air Miles per week fruit/vegetable intake self-reported). To further optimize the approach, a lottery could be layered on top of this assured incentive scheme, where participants who perform desired behaviors are entered into weekly draws to win additional Air Miles. This lottery feature could be used to exploit peoples’ tendency to overweight small probabilities [10] and drive target behaviors without significant additional resource. Notably, according to Klein & Karlawish, older adults may be more sensitive to lottery-based incentives than their younger counterparts [15].  Also, tailoring incentive offerings to demographic groups may boost enrollment and engagement. For example, making “meaningful” incentives more salient, by marketing iTunes credits to teenagers, wellness holiday credits to working adults, or grocery store vouchers to seniors, may increase the impact of relatively modest rewards. The newly developed Health Incentive Program Questionnaire may be useful in identifying preferred, more meaningful, and valuable voucher options (Mitchell et al, under review).

Though the Liu et al [9] paper is constrained by several limitations (eg, selection bias, sub-optimal incentive design, self-reported outcomes) it is an important launching pad for more sophisticated population-level evaluations of incentives for health. Incentives have emerged as a practical and acceptable public health policy alternative in Canada (and elsewhere), and so to promote broad, effective, and sustained implementation, future evaluations should stretch beyond the question, “Do incentives work?” to questions of moderations, such as, “Who or what behaviors are most sensitive to incentive intervention?”, “What types or sizes of incentives work best or are necesary?”, and “How long must incentives be in place to produce long-lasting change?”. Learning more about how to design incentives that produce clinically or health-economically significant changes, without being prohibitively costly, will only help to optimize this promising intervention in the future. 
